# Set1-mediated H3K4 methylation is required for *Candida albicans* virulence by regulating intracellular level of reactive oxygen species

**DOI:** 10.1080/21505594.2021.1980988

**Published:** 2021-10-26

**Authors:** Jueun Kim, Shinae Park, So Hee Kwon, Eun-Jin Lee, Jung-Shin Lee

**Affiliations:** a Department of Molecular Bioscience College of Biomedical Science, Kangwon National University, Chuncheon-si, Korea; b College of Pharmacy, Yonsei Institute of Pharmaceutical Sciences, Yonsei University, Incheon, Korea; c Department of Life Sciences, Korea University, Seoul, Korea

**Keywords:** *Candida albicans*, h3k4 methyltransferase, Set1, cellular ROS, virulence

## Abstract

*Candida albicans* is an opportunistic human fungal pathogen that exists in normal flora but can cause infection in immunocompromised individuals. The transition to pathogenic *C. albicans* requires a change of various gene expressions. Because histone-modifying enzymes can regulate gene expression, they are thought to control the virulence of *C. albicans*. Indeed, the absence of H3 lysine 4 (H3K4) methyltransferase Set1 has been shown to reduce the virulence of *C. albicans*; however, Set1-regulated genes responsible for this attenuated virulence phenotype remain unknown. Here, we demonstrated that Set1 positively regulates the expression of mitochondrial protein genes by methylating H3K4. In particular, levels of cellular mitochondrial reactive oxygen species (ROS) were higher in *Δset1* than in the wild-type due to the defect of those genes’ expression. Set1 deletion also increases H_2_O_2_ sensitivity and prevents proper colony formation when interacting with macrophage *in vitro*, consistent with its attenuated virulence *in vivo*. Together, these findings suggest that Set1 is required to regulate proper cellular ROS production by positively regulating the expression of mitochondrial protein genes and subsequently sustaining mitochondrial membrane integrity. Consequently, *C. albicans* maintains proper ROS levels via Set1-mediated transcriptional regulation, thus establishing a rapid defense against external ROS generated by the host.

## Introduction

*Candida albicans* is the most common fungal pathogen in humans. Although it is a member of the normal flora of the skin and mucosal surfaces in most healthy people, *C. albicans* can cause opportunistic infection in response to environmental changes in the host [1]. Indeed, *C. albicans* overgrowth can cause local or systemic infections, from candidiasis to life-threatening candidemia; therefore, it is necessary to study its pathogenesis to more effectively prevent and treat *C. albicans* infections [2].

When transitioning from a non-virulent commensal to a virulent pathogen, *C. albicans* undergoes various phenotypic switching events, including hyphal formation [1,3], white-opaque switching [4], biofilm formation [5], and secreted aspartyl proteinase expression [2,6–8]. In addition, *C. albicans* experiences a considerable change in the expression of genes encoding virulence factors, which are involved in recognizing the host environment [9]. Consequently, this leads to phenotypic switching and survival within the host, allowing *C. albicans* to proliferate rapidly by neutralizing and resisting host attacks [2,6,10]. Therefore, it is important to understand the differentially expressed genes and transcriptional regulation mechanisms that alter virulence-related genes’ expression.

One of the mechanisms that regulate transcription is the post-translational modification of histone proteins, which is a well-conserved phenomenon in all eukaryotes. Histone protein residues can be chemically modified in various ways, including acetylation, phosphorylation, methylation, and ubiquitination [11,12]. The methylation of histone H3 at lysine 4 (H3K4) is a well-conserved and widely studied histone modification due to its positive role in transcription [13,14]. In general, histone lysine residues can be mono-, di-, or trimethylated, and H3K4 trimethylation (H3K4me3) is a marker of active transcription given that it is enriched in the 5′ regions of actively transcribed genes [13,14].

A previous study revealed that the deletion of Set1, the only H3K4 methyltransferase in *C. albicans*, attenuated its virulence in mice [15]. H3K4 methyltransferase-deletion in other pathogenic fungi has also been shown to attenuate the virulence [16–21]. H3K4 methyltransferases positively regulate the expression of secondary metabolite genes in some fungal pathogens, thereby contributing to their virulence [17,20]; however, the mechanisms linking the effect of Set1 on virulence to transcriptional regulation by Set1-mediated H3K4 methylation in *C. albicans* remain unclear.

In this study, we investigated the effect of Set1 on the pathogenesis of *C. albicans* based on the finding that Set1-regulated genes are responsible for the pathogenicity of *C. albicans*. Whole-transcriptome sequencing (RNA-seq) revealed that the decreased expression of mitochondrial genes in Set1-deleted mutants increased cellular reactive oxygen species (ROS). Therefore, we suggest that *C. albicans* maintains proper ROS levels via Set1-mediated transcriptional regulation, thus establishing a rapid defense against external ROS generated by the host. Therefore, we suggest that Set1-mediated gene expression enables *C. albicans* to respond more rapidly to ROS generated by the host and protect against it.

## Materials and methods

### Strains and media

The *SET1* deleted strain (*Δset1*) was given from Dr Clancy group [15]. *C. albicans* strains were grown in YPD media (1% yeast extracts, 2% peptone, 2% glucose) at 30°C. The Raw264.7 mouse macrophage cell line was cultured in DMEM (Cellgro, 10–013-CV) with 10% FBS (Gibco, 26,140–079) and 1% penicillin/streptomycin (Gibco 15,140–122), in an atmosphere of 5% CO_2_ and 95% humidity at 37°C. For oxidative stress assay, overnight cultured cells were 10-fold serially diluted from starting OD_600_ of 0.5, and each 3 μl was spotted to YPD plate, including 5 mM H_2_O_2_ (Sigma, 216,763). The colony was observed after 2 days of culture at 37°C.

### RNA-seq analysis

Total RNA was extracted using NucleoSpin® RNA (MN, MN740955) according to the protocol of the manufacturer using each duplicated sample. *C. albicans* were grown in YPD and harvested at exponential phase (OD_600_ = 1.0). For sequencing, mRNA was captured using NEBNext® Poly(A) mRNA Magnetic Isolation Module (NEB, E7490), and a strand-specific sequencing library was synthesized using NEBNext® Ultra^TM^ Directional RNA Library Prep Kit for Illumina (NEB, E7420) according to the instruction manual. To compare the differential expression between wild-type (WT) and *Δset1*, we used DEseq2 normalization. Expression data was visualized using Heatmap generated by Pheatmap R package and Integrative Genomics Viewer (IGV) genome browser track.

### Chromatin immunoprecipitation (ChIP)

The ChIP assay was performed, as previously described [22]. Antibody used in the ChIP assay was anti-H3K4me3. ChIP DNAs were analyzed by quantitative PCR (qPCR) using the SYBR Green PCR mix (Toyobo, TOQPS-201) and the Applied Biosystems 7500 Real-Time PCR System. The sequences of primers used in this study are listed in Table S1.

### Cellular ROS observation

Overnight cultured cells were diluted to OD_600_ of 0.5 into fresh 5 ml YPD. After 1 h incubation at 30°C with shaking, cells were harvested and washed with PBS. For making spheroplast, cells were resuspended with zymolyase buffer (1 M sorbitol, 50 mM Tris-Cl, pH 7.4), added 10 μg zymolyase, and incubated at 30°C for 10 min. To observe cellular ROS, cells were incubated with 5 mM CellROX® Green (Thermo, C10444) at 37°C for 30 min, and sequentially DAPI (Sigma, D9542) was added and incubated for 10 min. After staining, cells were washed with PBS three times and observed under a fluorescence microscope.

### Macrophage interaction assay

For the survivability against macrophage attacks, harvested *C. albicans* cells were resuspended at 10^7^ cells ml^−1^ in cold PBS containing 10% FBS. RAW264.7 cells were seeded in each well of 96-well culture dish at 2.5 × 10^4^ cells 150 μl^−1^ per well or 5 × 10^4^ cells 150 μl^−1^ per well. Prepared *C. albicans* cells were serially diluted, and 50 μl cells were co-incubated with macrophages. The plate was incubated on ice for 30 min and followed by cultured for 24 h at 37°C in 5% CO_2_. After 24 h incubation, *C. albicans* colony was observed and counted to calculate survived cells.

### Mouse survival test

Animal experiments were performed at the Kangwon National University Animal Laboratory Center with approval through the Institutional Animal Care and Use Committee (IACUC) of Kangwon University (Approval Number KW-170302-5). Five-week-old female BALB/c mice were acclimated for 1 week, and tail vein injected with 10^6^ CFU of *C. albicans*. In 25 days after injection, survived *Δset1*-injected mice were euthanized with CO_2_ inhalation. Kidney, spleen, and liver sections were stained with H&E (Hematoxylin and Eosin) or PAS (Periodic Acid Schiff) and observed under a microscope.

## Results

### Set1 is required for full virulence but has a marginal effect on overall gene expression

**Figure 1.**
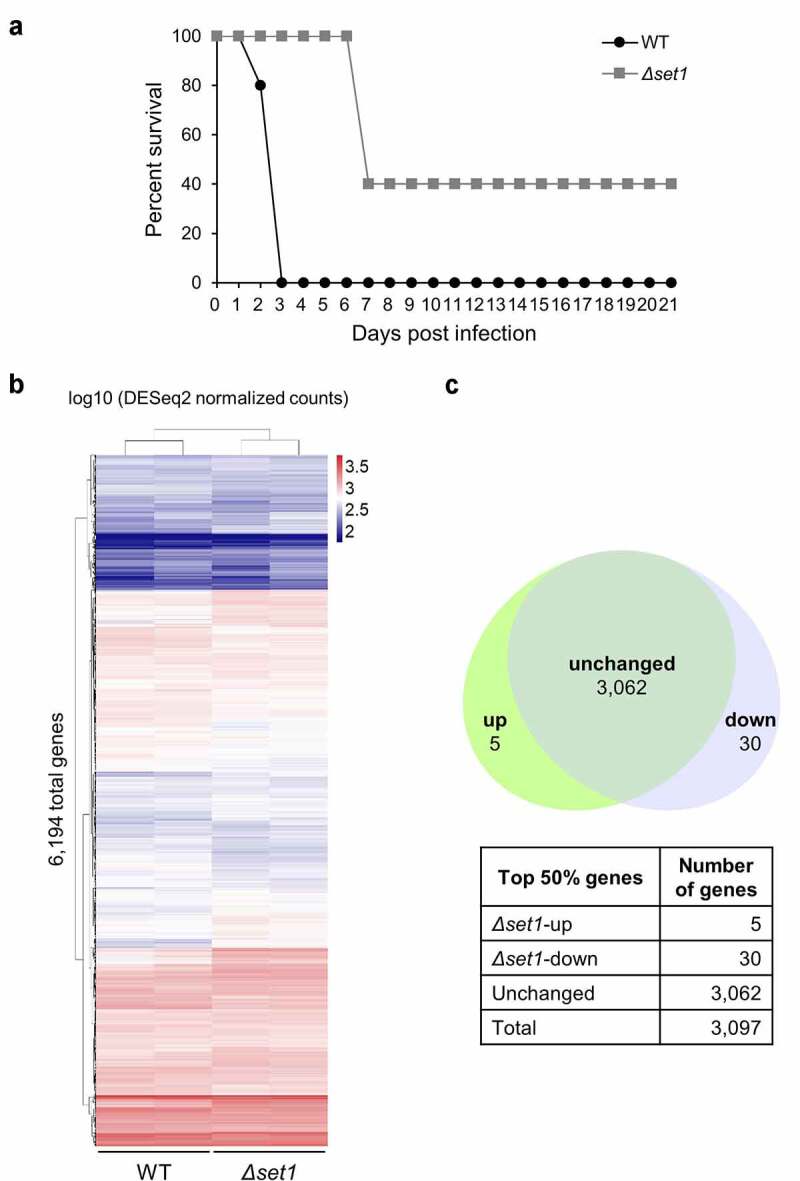
Set1 has little effect on overall gene expression although it is required for virulence of *C. albicans*. A, mouse survival analysis. the 6-week-old female BALB/c mice are injected intravenously with 0.1 ml of 10^7^ ml^−1^ cells. five mice were used for each strain. The *Δset1* strain has attenuated virulence in mice as reported by Dr Clancy group [15]. black closed circles, WT (SC5314, clinical isolated); Gray closed squares, *Δset1*. B, heatmap of total mRNA expression in WT and *Δset1*. Raw RNA-seq data were normalized by library size using DESeq2 and transformed into a log10 scale. the values were clustered and visualized using Pheatmap R Package. C, Venn diagram of top 50% genes with the highest expression levels. Top 50% expressed genes (3,097 genes in WT) were classified into three groups. Only 30 genes downregulated significantly in *Δset1* (adjusted *p* value < 0.05)

Set1 is the only H3K4 methyltransferase in *C. albicans* and is necessary for its full virulence in ICR mice [15]; however, the relationship between Set1-mediated H3K4 methylation and virulence remains unknown. Therefore, we investigated the mechanism by which Set1 regulates the pathogenicity of *C. albicans* through H3K4 methylation. First, we examined whether the virulence of *Δset1* was similar in BALB/c mice, which are inbred mice compared to outbred ICR mice. Briefly, two groups of mice (*n = *5 per group) were infected with 1 × 10^6^ CFU of wild-type (WT) and *Δset1 C. albicans* strains via tail vein injection, and their survival was observed for 21 days. We observed that BALB/c mice infected with *Δset1* survived longer than those infected with the WT (Figure 1(a)), indicating that the *Δset1* mutant displays attenuated virulence in this mouse model. Because the *Δset1*-infected mice that survived after 21 days appeared to be healthy, we examined the tissue condition of the infected but survived mice. No significant damage was observed in any *C. albicans*-infected tissues, including the kidney, spleen, and liver, compared to uninfected tissues. Moreover, *C. albicans* was not detected in any mouse tissues by staining with Periodic acid-Schiff (PAS), indicating that *C. albicans* had been cleared (Fig. S1).

For nonpathogenic *C. albicans* to become pathogenic in the host, large-scale alterations in gene expression are required. Previous studies have reported a positive correlation between H3K4 methylation mediated by Set1 and transcription [13,14,23]; therefore, we performed RNA-seq to determine which genes were differentially transcribed in *Δset1*. Surprisingly, we found that most genes’ overall expression patterns were unaltered, even without H3K4 methylation (Figure 1(b-c)). Moreover, several pathogenesis-related genes exhibited an increased expression in *Δset1* mutant (adjusted *p*-value <0.05) (Fig. S2A), contrary to our expectations from the defective virulence phenotype of *Δset1* mutant. Most of these genes with the increased expression in *Δset1* mutant were classified as cell wall proteins (Table S2).

**Figure 2.**
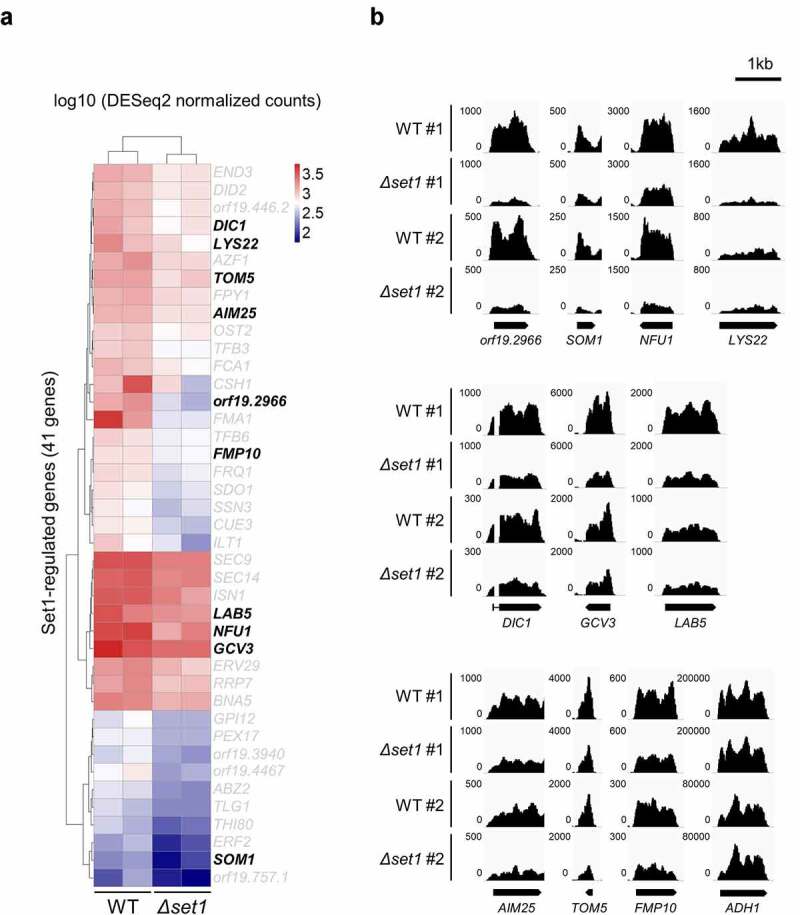
Set1 is required for proper expression of mitochondria-related genes and oxidative stress response genes. A, Heatmap of 41 Set1-regulated genes. The 41 genes are significantly downregulated in *Δset1* versus WT (adjusted *p* value < 0.05). Raw RNA-seq data were normalized by library size using DESeq2 and transformed into a log10 scale. The values were clustered and visualized as a heatmap using Pheatmap R Package. The mitochondrial genes are indicated in bold. B, RNA-seq data was visualized in Integrative Genomics Viewer (IGV) for 10 Set1-regulated genes related to mitochondria. *ADH1* gene is used as control. The y-axis range varies depending on the gene expression levels for each gene

We observed that only 3% of all annotated genes were differentially expressed in the absence of Set1 (adjusted *p-*value <0.05). In other words, the expression of 97% of genes was unchanged despite the lack of Set1-mediated H3K4 methylation. Remarkably, only 41 genes were downregulated in *Δset1* (Table S3 and Figure 2(a)), adjusted *p-*value <0.05). To allow the meaningful statistical analysis of expression levels, we selected the top 50% more highly expressed genes for further analysis (3,097 of 6,194 genes). Of these genes, only 30 genes showed a decrease in expression in the *Δset1* mutant (Figure 1(c), adjusted *p-*value <0.05). Because a previous study reported that Set1 can regulate pathogenicity by regulating adherence to host epithelial cells [15], we selected 100 genes with GO slim for adhesion among all *C. albicans* genes using the Candida Genome Database (http://www.candidagenome.org/) and checked the expression levels of selected 100 genes. As a result, we confirmed that the expression of most genes (93 of 100 genes) was not significantly different in *Δset1* mutant (*p*-value ≥0.05) (Table S4). In addition, adhesion of *C. albicans* is the first step in biofilm formation. When we checked the amount of biofilm formation through XTT assay, we did not observe significant differences in the amount of biofilm between WT and *Δset1* mutant (Fig. S2B).

It was previously reported that the expression of most genes does not change in the *Saccharomyces cerevisiae* strain lacking Set1 [24,25]; therefore, the authors of this genome-wide study suggested that Set1-mediated H3K4 methylation does not regulate gene expression [24]. Because the virulence phenotype was clearly reduced in *C. albicans Δset1*, we focused on the 41 genes whose expression decreased depending on H3K4 methylation. To examine the functional role of these downregulated genes in *Δset1*, we analyzed the functional annotation of each orthologous gene in *S. cerevisiae*, which is well studied in terms of functional genomics (Table S3). Although no gene was found to have virulence-related functions, functional annotation clustering (https://david.ncifcrf.gov/tools.jsp) analysis revealed that approximately 25% of the 41 *Δset1*-downregulated gene-encoded proteins were localized in the mitochondria, while most of the other gene products were located in the cytoplasm (Figure 2(b) and Table S3). Approximately 13% of the 6,100 total proteins in yeast cells are generally localized in mitochondria [26]; therefore, there were considerably more mitochondrial proteins among the Set1-dependent genes than average.

### Set1 regulates the expression of mitochondrial genes involved in biogenesis and protection against oxidative damage

The mitochondrion is the primary site for cellular ROS production since its inner membrane houses the electron transfer system (ETS), a series of four large complexes (I to IV) that transfer electrons from donors to acceptors via redox reactions and pump protons out of the mitochondrial matrix, forming a proton gradient [27–29]. Because this proton gradient drives proton-motive force, accumulated protons enter the matrix via ATP synthase to lower the proton gradient, leading to ATP synthesis. Electrons must pass through complexes to finally be absorbed as H_2_O, yet when electrons leak out, they react with O_2_ and become unstable superoxide anions (O_2_^−^•). Generally, O_2_^−^• is converted to hydrogen peroxide (H_2_O_2_) by superoxide dismutase (SOD), thereby reducing its reactivity; however, hydroxyl radicals (•OH) can be generated from H_2_O_2_ via the Fenton reaction and cause cellular damage through lipid oxidation, protein denaturation, and DNA mutations [27–29].

We hypothesized that the virulence attenuation observed in *Δset1* may be due to mitochondrial dysregulation. Thus, we focused on the 10 mitochondrial protein-coding genes, which were downregulated in the absence of Set1 in *C. albicans* (Figure 2(b)). The *S. cerevisiae* orthologs of the Set1-regulated genes, *SOM1* (orf19.6359) and *TOM5* (orf19.6247.1), play roles in the assembly and translocation of mitochondrial proteins, including the respiratory chain complex (Figure 2(a-b) and Table S3) [30–33]. A reduction in the expression of these genes could therefore reduce overall mitochondrial membrane integrity. Subsequently, the localization defect of the respiratory chains involved in oxidative phosphorylation could generate more mitochondrial ROS and eventually cause cellular damage. We also detected the downregulation of genes known to protect against oxidative stress damage in mitochondria, including *NFU1* (orf19.2067) and *AIM*25 (orf19.3929; Figure 2(a-b) and Table S3) [34–36]. We found that the expression of mitochondrial protein genes was downregulated in the absence of *SET1*, which may result in the production of many mitochondria-driven ROS. In addition, we found that the expression of some oxidative stress-responsive genes was downregulated (Table S3), suggesting that the *SET1-*deleted strain produces too much cellular ROS to remove, making *Candida* cells more sensitive to external attacks.

### H3K4 methylation is highly enriched in set1-regulated genes

**Figure 3.**
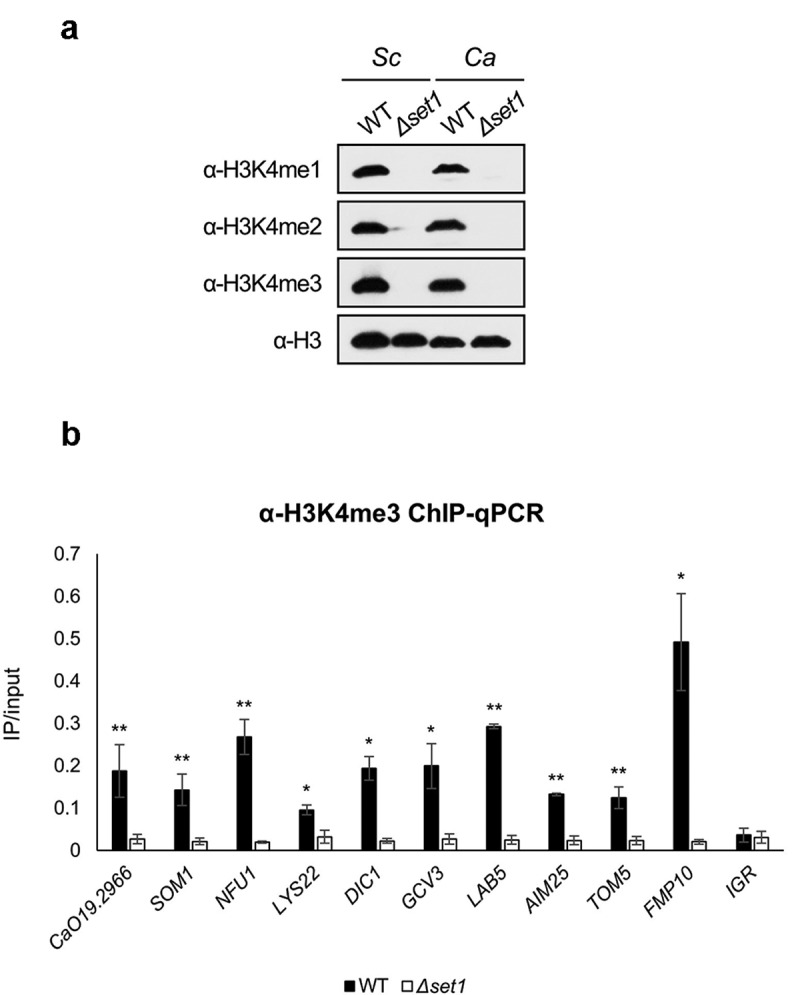
H3K4me3 is enriched in 5ʹ ORF of Set1-regulated genes. A, Western blot analysis of H3K4 methylation in *S. cerevisiae* (Sc) and *C. albicans* (Ca). FM391 is used as a WT control for *S. cerevisiae Δset1*. Set1 is the sole methyltransferase in *C. albicans*. Histone H3 is used as a loading control. B, H3K4me3 ChIP followed by qPCR was performed in WT and *Δset1*. The 5ʹ end sequences of Set1-regulated genes related to mitochondria were used as amplicon. Intergenic region (IGR) is used as a negative control. All ChIP analyses were performed in two independent biological replicates and qPCR was performed in triplicated. *p < 0.05 and **P < 0.01

Because H3K4me3 is an important histone modification for transcriptional initiation, we confirmed that Set1 mediated all three H3K4 methylation states using antibodies that recognize H3K4me1, -me2, and -me3, respectively (Figure 3(a)). We already selected 10 mitochondrial protein-coding genes whose expression decreased in *Δset1* strain (Figure 2(b)). We then carried out chromatin immunoprecipitation (ChIP) followed by qPCR (ChIP-qPCR) to determine whether these 10 mitochondrial protein-coding genes were regulated by H3K4 methylation. ChIP-qPCR with the H3K4me3 antibody revealed that H3K4me3 levels were high in the 5′ ORF of the mitochondrial protein-coding genes among the Set1-regulated genes in the WT (Figure 3(b)). Together, these results indicate that Set1 positively regulates target gene expression via H3K4me3, and that *SET1* deletion reduces target gene expression.

### Set1 deletion causes cellular ROS accumulation and renders Δset1 cells hypersensitive to external oxidative stress

**Figure 4.**
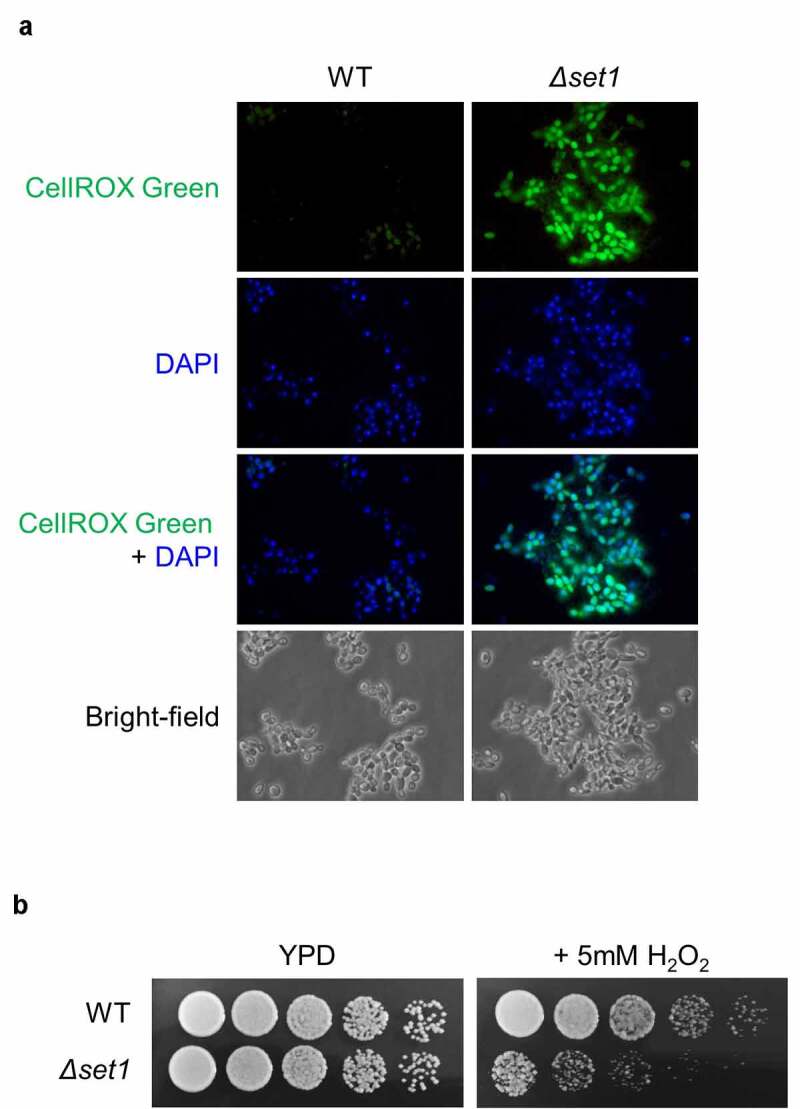
Cellular ROS is more generated in *Δset1*. A, Cellular oxidative status analysis in WT and *Δset1*. Cells were harvested in the early exponential phase and permeabilized with zymolyase followed by staining with 5 mM CellROX® Green. Cellular ROS was detected by fluorescence microscopy. C, Comparative growth of WT and *Δset1* on H_2_O_2_-containing media. Overnight cultured cells were diluted into 10^7^ CFU ml^−1^. 3 μl of 5-fold serial diluted cells were spotting on YPD or YPD added 5 mM H_2_O_2_. Each experiment was repeated at the same condition

We found that the expression of mitochondrial protein genes and oxidative stress response-related genes were downregulated in *Δset1* cells (Figure 2(a) and Table S4); therefore, we observed the cells under a fluorescence microscope to measure the levels of ROS generated using CellROX Green staining, which detects oxidative stress by binding to DNA when oxidized. Briefly, cells were treated mildly with zymolyase to produce spheroplasts for increased permeability and then stained with CellROX Green and DAPI. The CellROX Green signal was more potent in *Δset1* than in WT cells (Figure 4(a)), suggesting that the absence of Set1 increases cellular ROS levels.

We hypothesized that *SET1* deletion causes the mitochondrial membrane leakage, thereby accumulating cellular ROS and making cells susceptible to external oxidative stress. To test this hypothesis, we determined the sensitivity of the *Δset1* strain to oxidative stress by spotting them on H_2_O_2_-containing media. We observed that the *Δset1* strain was more sensitive to H_2_O_2_ than the WT strain (Figure 4(b)), consistent with our hypothesis.

### Set1-dependent gene expression is required for C. albicans survival in the host

Macrophages play a vital role in the innate immune system by degrading pathogens and sterilizing tissues [37]. When phagocytosis is induced by the host recognizing *C. albicans*, macrophages form inflammasomes and secrete ROS to attack the pathogen [38]. In response to the host, *C. albicans* expresses hypha-specific genes, resulting in morphogenesis and establishing a defense against oxidative stress [39]. Depending on the outcome of these processes, *C. albicans* is either cleared by macrophages or escapes from the phagosome and kills the macrophages [38]. To determine whether *Δset1 C. albicans* was more vulnerable to attack by ROS-releasing macrophages in the host, we performed an *in vitro* interaction analysis between mouse macrophages and *C. albicans*.

**Figure 5.**
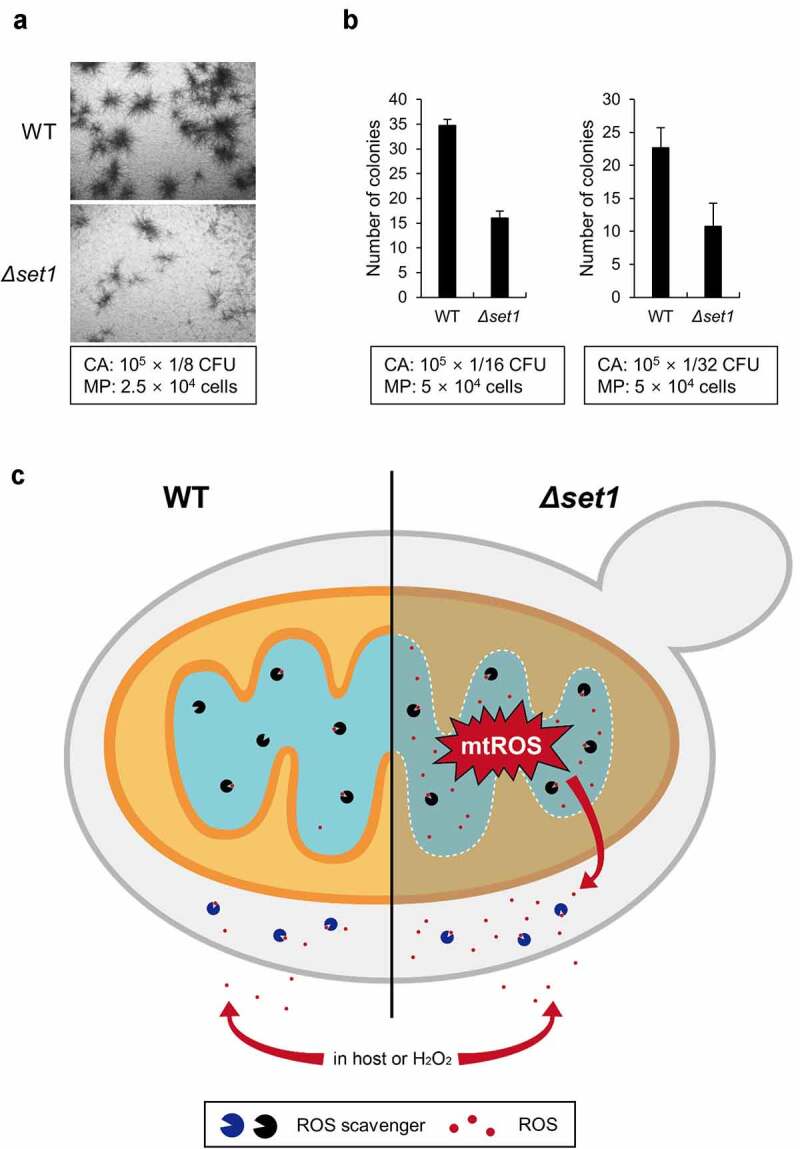
*Δset1* has attenuated virulence because of mitochondrial ROS leakage. A, The colony formation of *C. albicans* in the presence of RAW264.7 murine macrophage cells after 24 h incubation. The used amount of *C. albicans* and macrophage cells was represented in box. The representative microscopic images are shown. CA, *C. albicans*; MP, macrophage. B, The number of colonies was counted in the indicated ratio between *C. albicans* and macrophages and represented the average form. C, Proposed model. In WT, naturally occurring cellular ROS was cleared by ROS scavengers, represented as packman. On the other hand, because the mitochondrial protein genes are not properly expressed in *Δset1*, the mitochondrial membrane becomes loose and generates more cellular mitochondrial ROS than WT. Since the amount of ROS scavenger is the same as WT, the ROS accumulated in the cell is continuously neutralized by the antioxidant enzymes. However, if they are attacked by macrophage or treated with external ROS, *Δset1* is more susceptible to oxidative stress because of the already generated cellular ROS

To determine colony formation of *C. albicans* surviving macrophage attack, properly diluted *C. albicans* strains were cultured with the mouse macrophage cell line Raw264.7 (Figure 5(a)). We observed that the colonies formed by the *Δset1* strain were too small to be characterized as colonies, unlike those formed by the WT strain (Figure 5(a)). Moreover, the *Δset1* cells formed 50% fewer colonies than the WT cells (Figure 5(b)). These data suggest that Set1 contributes to *C. albicans* virulence by affecting resistance to and survival from macrophage attack. Because the virulence of *Δset1* is undoubtedly attenuated both *in vivo* and *in vitro*, we concluded that Set1 is required for the full virulence phenotype of *C. albicans* when infecting host tissues.

The antioxidant enzymes that protect cells from ROS are conserved in *C. albicans* including SOD, glutathione peroxidase (GPX), and catalase (CAT) [40–43] (*Candida* Genome Database; http://candidagenome.org/). The expression of antioxidant-encoded genes generally increases under oxidative stress conditions to neutralize ROS and prevent cell damage [44]; however, we found that the expression of these genes did not change in the *Δset1* strain, which contains high cellular ROS levels (Table S5), suggesting that Set1 deletion does not affect the ability to neutralize ROS. Because the *Δset1* strain contains high cellular ROS levels, its antioxidants are saturated faster than in the WT, making *Δset1* more vulnerable to ROS attack.

Overall, we propose that Set1 contributes to the virulence of *C. albicans* as shown in (Figure 5(c)). In WT cells, mitochondrial protein genes and oxidative stress response-related genes are generally expressed and localized correctly; therefore, mitochondrial ROS levels are low because WT *C. albicans* can immediately detoxify ROS as they are produced. Conversely, the mitochondrial proteins in *Δset1* are not correctly localized, causing electrons to escape from the respiratory chain and increasing mitochondrial ROS levels. Although the ROS scavenging system in *Δset1* eliminates ROS generated from the defective mitochondrial membrane, *Δset1* results in high ROS levels even in the absence of external oxidative stress. When *C. albicans* is attacked by an external ROS derived from host macrophages or is grown in a medium supplemented with H_2_O_2_, the WT strain can remove ROS under the oxidative stress, but *Δset1* cells cannot due to their saturated ROS scavenging system. Consequently, ROS quickly accumulates to a level that cannot be neutralized and decreases the survival rate of *C. albicans Δset1* as the cells are unable to resist oxidative stress (Figure 5(c)). Therefore, we propose that Set1-mediated gene expression is required for the survival of *C. albicans* against oxidative stress and its pathogenicity.

## Discussion

Opportunistic pathogens can become pathogenic via direct or indirect mechanisms: direct mechanisms involve a change in the ability of pathogens to attack hosts by altering their virulence factor expression, whereas indirect mechanisms involve pathogens passively enduring and surviving against attacks by the host immune system [45]. To understand why the virulence of *C. albicans* decreases in the absence of Set1, we identified genes regulated by Set1 using RNA-seq. The expression of genes directly involved in virulence did not change or was even upregulated in the *Δset1*strain; however, the expression of many genes required to resist oxidative stress, including mitochondrial protein genes, decreased significantly. The reduced expression of these genes increased the generation of mitochondrial ROS but did not reduce the growth rate compared to the WT under normal conditions (data not shown), since the generated cellular ROS could be neutralized continuously by antioxidant enzymes, such as SOD. However, the *Δset1* strain was more sensitive than the WT strain when treated with H_2_O_2_ or co-incubated with macrophages. This hyper-sensitivity was likely due to the inability of the *Δset1* strain to neutralize the excessive amounts of ROS generated during H_2_O_2_ treatment or co-incubation with macrophages. Indeed, the failure of the *Δset1* strain to induce oxidative stress-related genes in response to excessive ROS levels led to *C. albicans* cell death. These findings suggest that Set1 did not directly affect virulence by promoting the expression of virulence-related genes but instead lowered the production of mitochondrial ROS by regulating the expression of mitochondrial protein genes. Therefore, antioxidant enzymes remained available for external ROS attack to reduce ROS levels to normal physiological conditions. Together, these findings indicate that Set1 allows *C. albicans* to survive longer in the host and eventually switch to its pathogenic form (Figure 5(c)).

Histone H3K4 methylation and the H3K4 methyltransferase, Set1, are well conserved in many eukaryotes, and several studies have reported that Set1 and Set1-mediated H3K4 methylation are important for the pathogenicity of some fungal pathogens, in which Set1 regulates virulence and the stress response by controlling the expression of specific genes. For instance, Set1 has been shown to be involved in the virulence of the plant pathogen *Fusarium verticillioides* by regulating the expression of fumonisin B1 toxin-encoded genes [17], whereas Set1-mediated H3K4 methylation activates the expression of TR1, which encodes the toxin deoxynivalenol (DON), in the plant fungal pathogen *Fusarium graminearum* [20]. In addition, Set1 is involved in *Magnaporthe oryzae* fungal virulence by activating virulence-related genes via H3K4 methylation [21], while an H3K4 methyltransferase is required to induce genes in the entomopathogenic fungus *Metarhizium robertsii* under host conditions and induce virulence to mosquito infection [19]. Importantly, *C. albicans* is the only example that the virulent effects of Set1 have been reported in a human pathogen [15].

H3K4me3 is a marker of active transcription that is enriched in actively transcribed genes; however, we found that the absence of *SET1* did not change 97% of the total gene expression (Figure 1(a-b)). Similarly, recent genome-wide studies revealed that the absence of H3K4 methyltransferase did not dramatically change the overall gene expression in other fungal organisms, such as *S. cerevisiae* [46,47], *M. oryzae* [21], and *Fusarium fujikuroi* [18]. Because we observed that the expression of some genes increased in the absence of H3K4 methyltransferase, it is difficult to assume that the role of H3K4 methylation simply correlates with active transcription. Although the effect of Set1 was not significant in terms of the genome-wide expression profile, H3K4 methyltransferase has been implicated in the pathogenicity phenotype of various pathogenic fungi, including *C. albicans* [48]. In this study, we described the mechanism via which the absence of Set1 in *C. albicans* attenuates virulence, with a particular focus on the genes regulated by H3K4 methylation. Transcriptome analysis revealed that RNA levels did not change significantly in *C. albicans* in the presence or absence of Set1 under normal conditions; however, there may be a set of genes whose expression differs depending on the presence or absence of Set1 under oxidative stress conditions or during interactions with macrophage. Therefore, the role of H3K4 methylation in transcriptional regulation cannot simply be defined as active or negative regulation, but must be described as a function that regulates each gene differently in specific environments and should be investigated in future studies.

## Supplementary Material

Supplemental Material

## Data Availability

Sequencing data sets generated and/or analyzed during this study have been deposited to the Gene Expression Omnibus Database (GEO) under the accession number: GSE158481 (https://www.ncbi.nlm.nih.gov/geo/query/acc.cgi?acc=GSE158481). All data generated or analyzed during this study are included in this published article and its Additional files.
